# It’s What’s True

**DOI:** 10.1093/oncolo/oyac241

**Published:** 2022-11-25

**Authors:** Benjamin L Sievers

**Affiliations:** Infectious Diseases, J. Craig Venter Institute, La Jolla, CA, USA

## Abstract

A cancer survivor describes the journey from healthy student through diagnosis and treatment for thyroid cancer and back again

“It’s what’s true,” I remember my oncologist father’s words when an unusually friendly nuclear medicine technologist wearing a beanie with flaps above his ears enters my room, wheeling a cart holding a large lead pig. “Ben, can you spell for me your last name and give me your date of birth, please?”

I had believed myself to be a healthy 22-year-old student graduating from college, studying to take the MCAT, and planning my Fulbright research fellowship in Phnom Penh. Now, I have thyroid cancer.

I first knew something was wrong when a long strap muscle in my neck snapped over an enlarged lymph node just above my clavicle as I turned my head to merge lanes. A week later, my new surgeon gently plunged a needle into my neck as I stared at various shades of gray on an ultrasound screen. I remained utterly still as we all visualized the needle tip on the screen, and I was scared. At the same time, I felt considerable resolve. My tumor cells smeared on a microscope slide would illuminate truth and define next steps. Together, we’d learn “what’s true.”

Papillary thyroid carcinoma was confirmed from the needle aspiration procedure and a spontaneous cancellation offered an auspicious opening in my surgeon’s busy schedule, enabling me to have my cancer procedure that same week. My concerned parents seemed pleased to move quickly. Me? A bit more reluctant. Although looking back, I now don’t regret missing weeks of anxious anticipation. The night before my procedure, my family and I shared a quick meal and tried to cultivate some humor and a bit of perspective. “My surgeon said I had stage I disease because I’m young. I guess that’s a plus, right?” My family’s heads nodded affirmatively. Upon awakening the next morning, curiosity and more resolve were what I felt in the 5 AM quiet darkness of the preoperative waiting room as I waited for me to be summoned. “Let’s do this.”

I experienced exceptional kindness from everyone I met that morning. My IV was placed with competent flourish and an easy humor seemed to flow among the OR nurses. I appreciated this greatly. I later learned that my family walked loops in the hospital parking lot for the four hours as I received a complete thyroidectomy and modified radical neck dissection. While I don’t remember much immediately post-op, I awakened with thick plastic tubes in my neck that moved when I swallowed or spoke. Almost immediately, I was desperate to get mine out. Gratefully, in my post-op clinic with a gentle tug from my surgeon, my tubes slid out like tired snakes. Now, an “epic” scar traces from just under my ear to the middle of my throat.

And now, my formal diagnosis of follicular variant papillary thyroid carcinoma was established with 6 of 38 nodes infiltrated by cancer. The pathologist noted “tall cells” that are believed to be somewhat less likely to take up radioactive iodine. I underwent staging and I also have three sub centimeter nodules in my lungs of “unclear significance.” Call these metastases, and that’s not good, but call them post-infectious granulomas, and that’s sweet reassurance. Next-generation sequencing of my tumor identified an ALK fusion gene. Reading more, I learned that not much was known about its significance in thyroid cancer, although there were case reports suggesting an association with more aggressive disease. At the same time, ALK inhibitors in clinical trials were anecdotally revealing promising outcomes. Here we were again: reasonably good news mixed with challenging news. Ultimately, my physicians celebrate my youth because it’s associated with a good overall prognosis, despite my cancer’s high-risk features. That’s also “what’s true.”

I then met in consultation with a radiation oncologist, who recommended a 150 mCi dose of radioactive iodine aimed at eradicating any residual cancer. I wanted to know the basis of this guidance, and learned it was not based on any randomized trials. Like many medical practices, the relative dose levels were prescribed based upon an increasing risk of cancer recurrence from historical outcomes. Different doses of radioactive iodine were used based upon a broad consensus. I didn’t feel that this was wrong, but at the same time, it surprised me that there had not been more clinical trial efforts to clearly define optimal dosing for patients like me.

With the risk of secondary malignancy and salivary gland damage increasing linearly as higher radiation doses were administered, I wanted to better understand what an optimal dose might be for me, given how many of my nodes were cancerous. I scoured the internet and spoke with international experts. Some were reassured by my decreased blood tumor markers and made the case that a lower dose now could always be followed by additional therapy in the future if needed. Conversely, my local care team recommended a higher dose of radiation because that had “always been” their approach. Opinions varied, and I wrestled with this vexing dosing decision. My oncologist father was particularly anguished with the lack of data to guide such an important element of my treatment plan.

Ultimately, I agreed to a treatment course using 100 mCi. To me, it was the dose that balanced the potential benefit to address the risk of recurrent disease against the side effects of chronic salivary gland damage and future potential of a secondary cancer. I resigned myself to the possibility of taking another swing at radioactive iodine if I have persistent cancer.

Try as I might, I struggle to accept the idea that my cancer places me at a high risk of recurrence. Sometimes I think to myself, maybe it could have been different if I had noticed it sooner. Instead of a 9-inch scar, I would have a 2-inch scar. I’m chagrined that I didn’t notice this Oreo-sized, rubbery, painless disc earlier. But I suppose all of us with cancer could share similar bewilderment stories capturing the instant we *knew*.

What do I understand now about how thyroid cancer is approached? For those who have to decide how best to treat us, previous patients, their presentations, and their pathology creates their “visceral memory.” Particularly challenging anecdotes of risk followed by recurrence can stay with thyroid thought leaders. Without randomized trial data to inform the optimal dose of radioactive iodine to deliver as an “adjuvant” treatment, I’m witnessing considerable art in the practice of my medicine.

Where medical approaches veer from a well-traveled highway to a dirt road—this is what I feel my journey has been like. And for me, the consequences of clinical uncertainty and anecdotal medicine continues to impact my life as well as my future as a physician.

And I’m now in the Orussey live bird market in the heart of Phnom Penh, Cambodia. The air is thick and packed with grayish-white feathers and dust. Hundreds of birds wrestle their red rope restraints on wooden pallets that line the cracked stone floor under the tin-roofed enclosure. Mud, blood, and water cake our shoes. We stand close, hovering a small AeroCollect air sampler over a bubbling pot of freshly de-feathered ducks. Later, in the lab, we learn that these air samples have now tested positive for H5 avian influenza RNA.

I’m a thyroid cancer survivor living my very best life, and this is what’s true for me.

